# Measurement of Variations in Gas Refractive Index with 10^–9^ Resolution Using Laser Speckle

**DOI:** 10.1021/acsphotonics.1c01355

**Published:** 2022-02-16

**Authors:** Morgan Facchin, Graham D. Bruce, Kishan Dholakia

**Affiliations:** † SUPA, School of Physics and Astronomy, University of St Andrews, North Haugh, St Andrews KY16 9SS, United Kingdom; ‡ Department of Physics, College of Science, Yonsei University, Seoul 03722, South Korea; § School of Biological Sciences, The University of Adelaide, Adelaide 5005, South Australia 5005, Australia

**Keywords:** speckle patterns, metrology, integrating sphere, refractometry

## Abstract

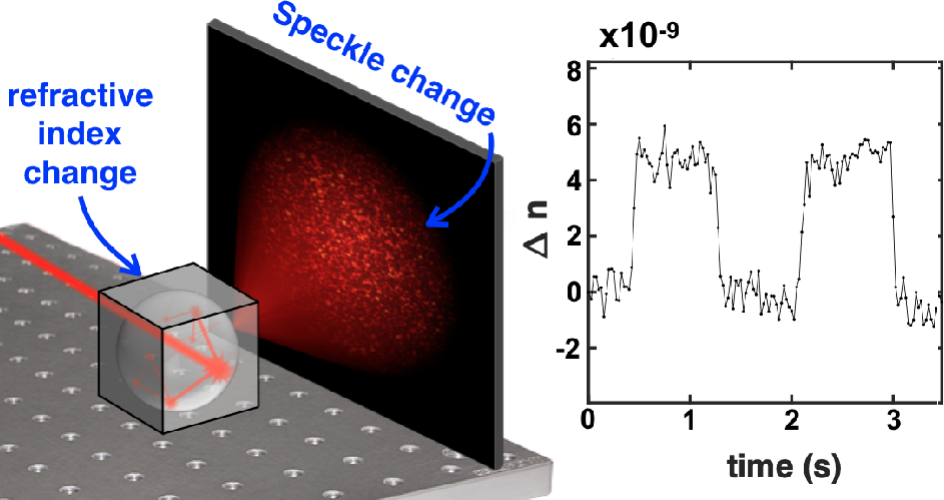

Highly resolved determination of refractive index is vital in fields ranging from biosensing through to laser range finding. Laser speckle is known to be a sensitive probe of the properties of the light and the environment, but to date speckle-based refractive index measurements have been restricted to 10^–6^ resolution. In this work we identify a strategy to optimize the sensitivity of speckle to refractive index changes, namely, by maximizing the width of the distribution of optical path lengths in the medium. We show that this can be realized experimentally by encapsulating the medium of interest within an integrating sphere. While mitigating against laser-induced heating effects, we demonstrate that variations of the refractive index of air as small as 4.5 × 10^–9^ can be resolved with an uncertainty of 7 × 10^–10^. This is an improvement of 3 orders of magnitude when compared to previous speckle-based methods.

Refractive index is a parameter of importance across most areas of optical measurement. It can be used in cell biology to investigate particular cell metabolic activities or as a probe of other biophysical quantities,^1^ and in chemical sensing, it can be used to measure concentrations of liquids.^2^ Interferometric measurements of length and displacement in gaseous environments^3^ are limited in their accuracy by uncertainties in the refractive index of the environment. High-precision measurements of refractive index have even been proposed as a route to a more accurate definition of the Pascal.^4^ Small changes in refractive index can have major implications: Infection can cause the refractive index of red blood cells to change at the 10^–3^ level;^5^ biosensors measure cell secretion dynamics and protein concentrations by tracking refractive index changes at the 10^–5^ level.^6,7^ In optical tweezers experiments exploring the motion of RNA polymerase during transcription,^8^ the effect of air currents (which typically modulate the refractive index at the order of 10^–7^)^9,10^ caused sufficient position instability of the optical trapping and measurement beams to mask the Å-level motion, even in a sealed environment. There are many methods to measure refractive index using lasers, including hollow core,^11^ photonic crystal,^12,13^ or evanescent optical fiber refractometers^14^ (fiber-based devices have been recently reviewed in ref (15)) and metasurfaced-based refractometers.^7^ The most sensitive measurements of refractive index in the literature are variants of double-channel Fabry–Perot cavities,^10^ with which refractive index uncertainties of 10^–12^ have recently been demonstrated.^4^

Laser speckle, formed when a coherent light field interacts with a disordered medium, is a powerful probe of changes to the laser or the medium itself and is, therefore, an attractive tool to harness sensing applications. The first application of speckle in refractometry was presented half a century ago,^16,17^ and most subsequent work has adopted a similar scheme. A laser beam impinges on a random phase screen to produce a speckle field, which then traverses a medium under investigation, and the changes in the speckle pattern can be used to quantify changes in the refractive index. Speckle has been applied to measurements of the refractive indices of air,^18^ glass,^19^ and liquids.^20^ Recently, by immersing up to three consecutive planar diffusers inside a medium of interest, Tran et al. used the resultant speckle to measure the refractive index with a resolution on the order of 10^–6^.^21^

How might one further optimize the sensitivity of a speckle refractometer? A speckle pattern is the result of the interference of many different wave paths. When light propagates in a medium of refractive index *n*, the phase acquired on a given path of length *z* is *nkz*, with *k* being the wavenumber. A change in the refractive index therefore applies a phase shift Δ*nkz* on that path. Now, any change occurring in the speckle pattern results from relative phase changes between paths. It follows that what maximizes the sensitivity of the speckle pattern is not the path length itself, as intuition might suggest, but the spread of the path lengths within the medium of interest. In a simple illustrative case where we consider only two given paths, their relative phase changes by *k*Δ*n*Δ*z*, where we see that the maximal effect is obtained for a maximal path length difference. This strategy is consistent with the work of Tran et al.,^21^ where the speckle sensitivity is greatly increased by the use of successive planar diffusers inside the medium, increasing the number of paths and their length differences. However, that approach is still limited by the globally paraxial geometry of the diffusion.

In this work, to further increase the spread of the path lengths and enable more finely resolved measurements of refractive index, we take inspiration from recent progress in speckle-based measurements of the wavelength of monochromatic light.^22−28^ In that context, we have recently analytically shown the advantages of using an integrating sphere to generate the speckle.^29^ The integrating sphere creates a particularly broad path length distribution that offers orders of magnitude improvement in sensitivity compared with other speckle-based techniques.

To quantify the change in the speckle due to a refractive index change, we develop a method based on the evaluation of the speckle similarity. This speckle similarity is then used to extract the refractive index change, based on an explicit relation existing between the two, that we derive analytically and verify experimentally. By varying the pressure of air inside an integrating sphere, we resolve changes in the refractive index as low as 4.5 × 10^–9^ with an uncertainty of 7 × 10^–10^. We also discuss the role of laser-induced heating and identify strategies for its compensation. Our method surpasses all previous speckle refractometers and has the potential to match the existing state-of-the-art measurements of small changes in the refractive index of gases.

## Similarity Profile

The quantitative tool that we use in this work is the speckle similarity, or correlation, which quantifies the change occurring in a speckle pattern. It is defined as
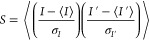
1where *I* and *I*′ are two speckle images (before and after a change), σ_*I*_ and σ_*I*′_ are their respective standard deviation, and the angular brackets denote averaging over the image. This gives a value of 1 for identical images and decreases toward 0 as they diverge from one another.

It was shown in ref (29) that, for two speckle patterns taken before and after some generic transformation, the similarity is given by
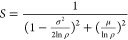
2where μ and σ^2^ are, respectively, the mean and variance of the phase shift induced by the transformation on a single pass of light through the sphere, with ρ being the sphere’s surface reflectivity.

Let us apply this to the case of a change in the refractive index of the medium filling an integrating sphere of radius *R*. The phase light acquires on a given path of length *z* is *nkz*, with *n* being the refractive index and *k* being the wavenumber. After a refractive index change, the phase changes by an amount Δ*nkz*. It follows that the average phase change on a single path is μ = Δ*nkz̅*, with *z̅* being the average chord length in the sphere, which is given by geometry to be 4*R*/3.^30−32^ Likewise, the standard deviation of chord length is √2*R*/3.^31,32^ This gives
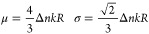
3

Inserting this into eq 2, it can be shown that the μ term dominates, which leaves us with a Lorentzian profile:
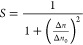
4with Δ*n*_0_ = 3λ|ln ρ|/(8π*R*), which also corresponds to the HWHM of the Lorentzian. For modest parameters such as *R* = 1 cm, ρ = 0.9, and λ = 780 nm, this gives Δ*n*_0_ = 10^–6^.

This similarity profile can be used in a very simple way to determine the refractive index difference Δ*n* between two given times by taking the reciprocal function
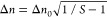
5with *S* being the similarity between the two corresponding speckles. As we will show, refractive index changes much smaller than Δ*n*_0_ can be resolved; the ultimate resolution of the method mainly depends on the wavelength noise and detector noise.

## Experimental Implementation

**Figure 1:**
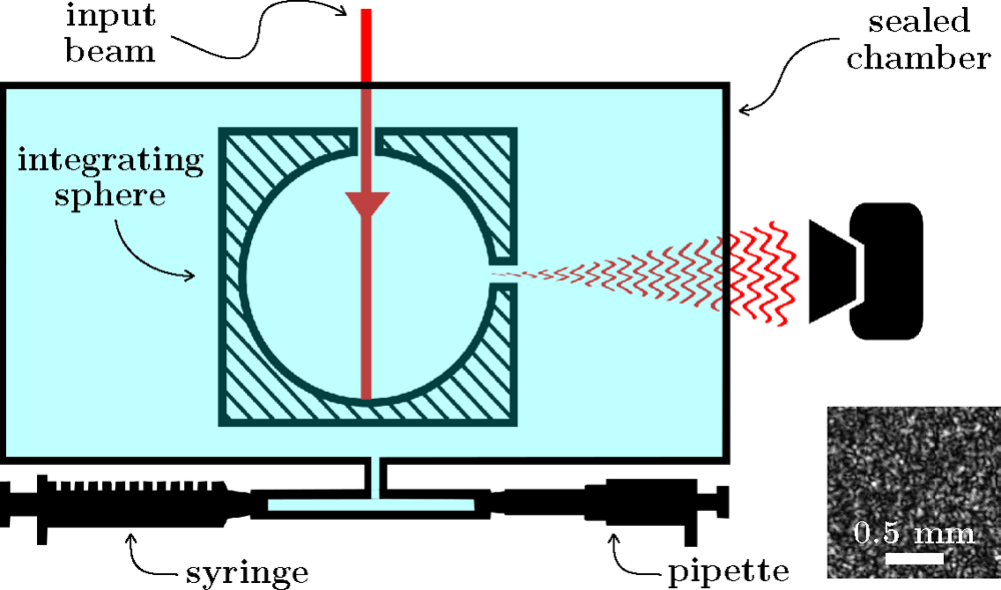
Experimental setup. An integrating sphere is placed in a sealed chamber where light can enter and exit via two glass windows. The air inside the chamber is slightly compressed by pushing a syringe (for the verification of eq 4) or a pipet (for the measurement of small variations). The resulting change in refractive index of the air induces a change in the speckle pattern that is recorded on a camera. An example of the resultant speckle pattern is shown.

In this section, we experimentally verify eq 4, with the setup described in Figure 1. A laser beam of wavelength 780 nm, 10 mW of power, and a coherence length of a few kilometers (Toptica DLPro) is injected into an integrating sphere, and the resulting speckle pattern is collected on a CMOS camera (Mikrotron MotionBLITZ EoSens mini2). We use a 1.25 cm radius sphere that is carved into a 3 cm edge aluminum cube and coated with Spectraflect to make a Lambertian and highly reflective surface. The light enters and escapes the sphere through two 3 mm diameter holes. The sphere is placed in a 2490 ± 50 mL stainless steel chamber that is hermetically sealed using CF flanges and copper gaskets.

**Figure 2:**
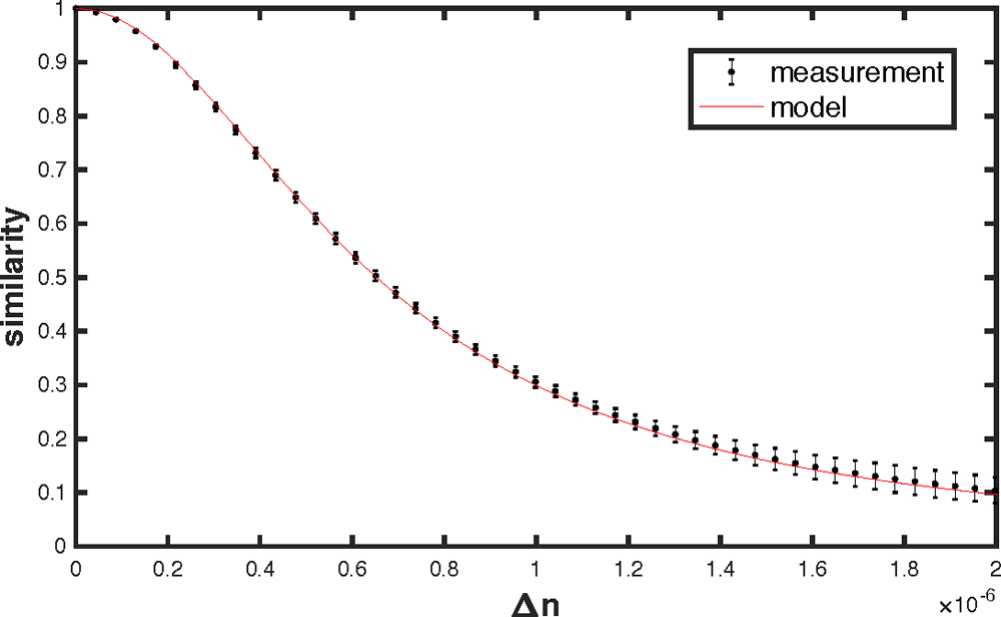
Speckle similarity as a function of refractive index change, experimental (black dots) and Lorentzian profile predicted by model (red line), fitted for a reflectivity ρ = 0.916. The center and span of the error bars, respectively, give the mean and standard deviation of a set of curves extracted from the data set. The HWHM is 6.5 × 10^–7^.

The refractive index variations are obtained by slightly compressing the air inside the chamber using a 100 mL syringe connected to the chamber via a needle valve. The syringe is compressed at a constant rate of 4.0 mL s^–1^ using a motorized translating stage while the changing speckle is recorded. From this we extract the similarity profile as a function of refractive index change shown in Figure 2. The value of the refractive index change is inferred from the volume change by the following. The fact that the chamber is sealed implies Δ*n*/*n*′ = Δρ_air_/ρ_air_ ≈ −Δ*V*/*V*, with *n* = 1 + *n*′ being the refractive index of the air inside the chamber, ρ_air_ is its density, and *V* is the chamber’s volume, assuming *n*′ ∝ ρ_air_ (Gladstone–Dale law^33^) and Δ*V* ≪ *V*. It follows that the refractive index change is given by Δ*n* = −*n*′Δ*V*/*V*, with *n*′ = 2.7 × 10^–4^ for our measured values of λ = 780 nm, 20 °C, and 100.5 kPa.^34^ The main source of uncertainty is the volume of the chamber (2%). In Figure 2 we also display the uncertainty of the profile, given by the standard deviation of a set of curves extracted from the data set by using different reference images. By fitting the resulting profile using eq 4, with the reflectivity as a free parameter, we find the best agreement for ρ = 0.916 ± 0.002, which is consistent with our previous estimations.^29^ The HWHM is found to be Δ*n*_0_ = 6.5 × 10^–7^, corresponding to a volume variation of only 6.0 mL or a fractional volume change of 0.24%. This can be done very easily by hand without feeling any pressure resistance, which offers a very hands-on demonstration of speckles’ sensitivity.

## Measurement of Small Refractive Index Changes

In this section we describe the measurement of refractive index variations that are much smaller than Δ*n*_0_. For such variations, using eq 5 directly is not ideal; as for Δ*n* ≈ 0, we have d*S*/dΔ*n* ≈ 0. This problem would be solved if we could look at small variations of the similarity around its point of maximal slope, which occurs at Δ*n* = Δ*n*_0_, instead of around Δ*n* = 0. This, in fact, can be done by purposely applying an initial refractive index variation of Δ*n*_0_ prior to the measurement. In this way, the similarity, taken between a speckle before and after the initial Δ*n*_0_ leap, varies around a value of 0.5 with maximal sensitivity. In our setup, this initial variation could be applied by changing the volume of the chamber by 6.0 mL. However, a simpler way is to make use of the equivalence that exists between refractive index variation and wavelength variation. Specifically, the phase shift resulting from a wavelength change on a path of length *z* is *n*Δ*kz*, which is of the same form as what we found for a refractive index change (Δ*nkz*). As both phase shifts are proportional to *z*, equating them on one path equates them on all paths, and the two effects are physically equivalent when Δ*n* = *n*Δ*k*/*k* ≈ −Δλ/λ (with *n* ≈ 1 and Δλ small). This means that the same change in a speckle pattern occurs after a refractive index change Δ*n* or after a wavelength change Δλ = −λΔ*n*. We can therefore bring the similarity to its point of maximal slope by applying an appropriate wavelength offset, in our case, equal to 0.5 pm.

**Figure 3:**
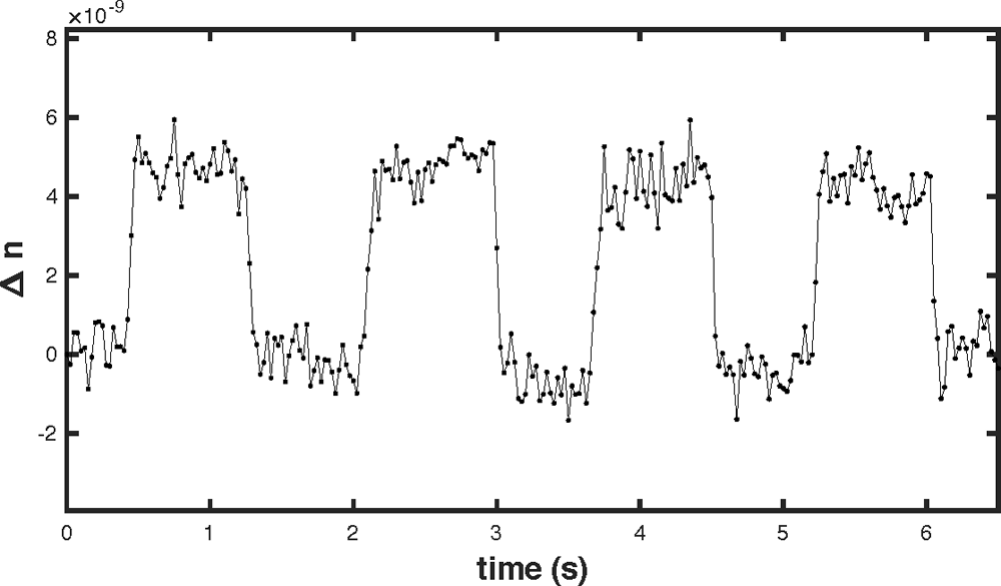
Measurement of small periodic steps in the refractive index, applied by changing the volume of the chamber by 40 μL using a pipet, corresponding to a fractional volume change of (1.61 ± 0.03) × 10^–5^. We find a step amplitude of (4.5 ± 0.7) × 10^–9^, in accord with the expected value of (4.3 ± 0.1) × 10^–9^.

We proceed in the following way. A reference speckle is first recorded at an initial wavelength. The wavelength is then offset by about 0.5 pm (this does not need to be precise). Thereafter, small refractive index changes are applied using a pipet instead of a syringe, which can apply much smaller volume changes. We press and release the pipet in a square wave manner with a period of about a second, with a 40 μL volume load. This corresponds to a fractional volume change of (1.61 ± 0.03) × 10^–5^, from which we infer an expected refractive index change of (4.3 ± 0.1) × 10^–9^. We compute the similarity between the reference speckle and the speckles undergoing change, which is then converted to refractive index difference using eq 5. The resulting curve is shown in Figure 3. The first value of the time series is subtracted, so that what is displayed is the refractive index variation applied by the pipet. We find steps of amplitude (4.5 ± 0.7) × 10^–9^, which is consistent with the expected value.

## Uncertainty

As the slope of the similarity profile at the inflection point is 1/(2Δ*n*_0_), we can express any small refractive index variation as δ*n* = 2Δ*n*_0_δ*S*, where δ denotes a small variation around the inflection point. For estimating our measurement uncertainty, we also include laser wavelength fluctuations, as this sets a fundamental limit to performance. We now have
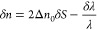
6where we used the fact described above that refractive index variations are equivalent to wavelength variations when Δ*n* = −Δλ/λ. From this we infer the uncertainty relation
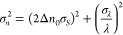
7where σ denotes the uncertainty (standard deviation of the noise) on each quantity. σ_*S*_ is dominated by the Poisson noise on each individual pixel, which propagates to the estimation of the similarity, and σ_λ_ is a property of the laser source used. Fluctuations in the input beam polarization, position, or angle produce only negligible contributions to the noise, as the similarity of speckle from an integrating sphere is largely independent of path-independent effects.^29^ We are then facing two possible cases, where either the σ_*S*_ or the σ_λ_ term dominates.

When σ_*S*_ dominates, which is the case in our experiment, we have σ_*n*_ = 2Δ*n*_0_σ_*S*_. In principle, σ_*S*_ could be determined analytically knowing the probability law of the noise on each pixel and the explicit expression of the similarity, but this turns out to be a very difficult problem. Instead, we find an empirical law for σ_*S*_, approximately given by 0.1/√*N* for our camera and illumination conditions, with *N* being the number of pixels (for our image size, 200 × 200, this gives σ_*S*_ ≈ 5 × 10^–4^). Inserting the expressions of Δ*n*_0_ and σ_*S*_, we find
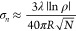
8With our parameters, this gives σ_*n*_ = 7 × 10^–10^, which is in accord with the level of noise found in Figure 3.

As σ_*S*_ is reduced by increasing image size, the σ_λ_ term may become dominant, which sets a lower limit to performance with an uncertainty σ_*n*_ = σ_λ_/λ. Note that estimating the similarity noise as a function of image size can serve as a way to measure σ_λ_. Indeed, if a plateau is reached as the image size is increased, σ_λ_ can be inferred from the value of that plateau.

In this work, we use a laser with low wavelength noise (less than 0.1 fm) and a standard CMOS camera. Therefore, we are in the first case described above where camera noise dominates. However, this cannot be the reason for our lower uncertainty compared to other reported works. Indeed, the best performance found in the literature uses a HeNe laser,^21^ and if the reported uncertainty (2 × 10^–6^) were to be attributed to wavelength noise, we would have σ_λ_ = 3 pm, which is much higher than the typical fm-level noise of a wavelength-stabilized HeNe laser. As the effect of camera noise increases with the HWHM, it is probably the case that this is the dominant source of uncertainty in most other works reported in the literature.

## Heating Effect and Compensation

**Figure 4:**
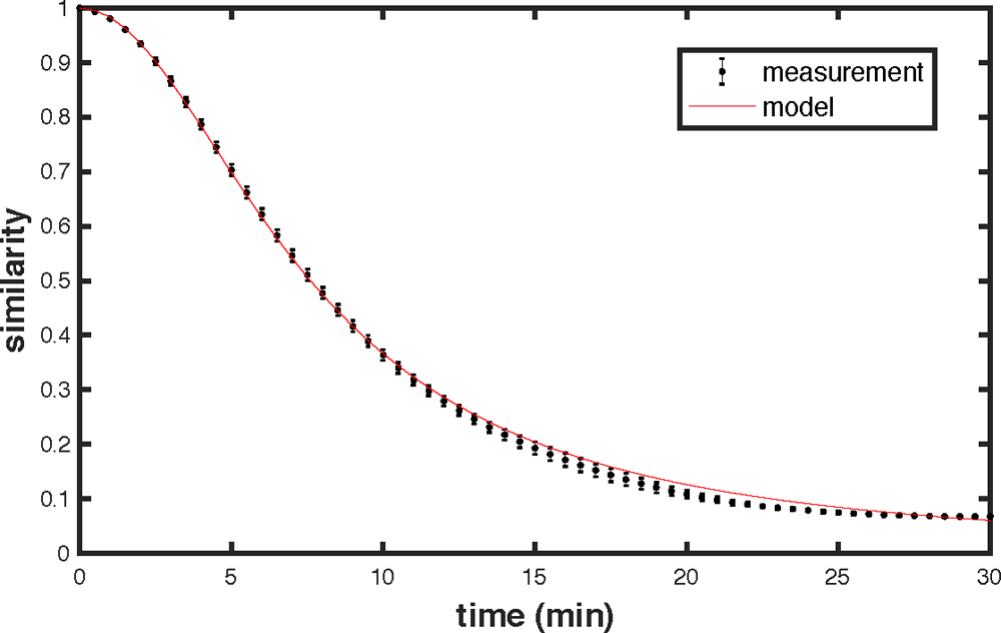
Intrinsic change in the speckle pattern over time when no transformation is applied, caused by heating from the laser. Black: similarity between the speckle at a reference time and subsequent times. Red: Lorentzian fit with a HWHM of 7.6 min. We can infer from this that the radius of the sphere increases by 1.1 nm every minute.

When the system described in Figure 1 is left on its own without applying any transformation, we still observe a slow change in the speckle pattern over time. When quantifying this change by the similarity, we obtain the curve shown in Figure 4, which (surprisingly) is also well fitted by a Lorentzian profile, with a HWHM of 7.6 min. We make the hypothesis that this time evolution comes from heating due to the input light. Inside the sphere, the diffusion of light is such that the surface power density is uniform across the inner surface. Therefore, in a steady state, nearly all the input power is absorbed uniformly on the inner surface (the power of the escaping light is negligible). The heat can then either be conducted to the material or to the air inside the sphere.

In the case of the material, an order of magnitude estimation shows that the heat diffusion time is very short (of the order of seconds) so that we can assume the increase in temperature to be uniform throughout the material. This temperature increase, in turn, leads to an isotropic thermal expansion of the sphere. The effect of such an expansion can be found analytically: the phase acquired by the field on a given path of length *z* being *nkz*, and as an isotropic expansion increases all lengths by a factor Δ*R*/*R* (with Δ*R* the variation of radius resulting from the expansion), the resulting average phase shift on a single pass is 4*k*Δ*R*/3 (with *n* ≈ 1). Assuming in a first approximation that the heat remains stored in the sphere’s material, we have Δ*R* ∝ *t*, which inserted in eq 2 indeed leads to a Lorentzian profile in time. One could expect that the increase in temperature of the sphere’s material in turn induces a heat flux from the sphere to the surrounding air, leading to thermal equilibrium and a stop to the thermal expansion. However, we observed that the HWHM of the Lorentzian does not change significantly in over 80 min of measurements, meaning that no thermal equilibrium is reached in that time. This indicates that the approximation of the heat remaining in the sphere’s material is valid at least in that time scale. Given that after a time equal to the HWHM we have the relation 4*k*Δ*R*/3 = ln ρ, we can infer that the radius of the sphere increases by 8.1 nm every 7.6 min by thermal expansion, which corresponds to a speed of 1.1 nm·min^–1^ or 18 pm·s^–1^.

We also see that, as the phase shift due to thermal expansion has the same form as that of a refractive index change, we can also draw an equivalence between those two effects, given by the substitution Δ*n* ⇔ Δ*R*/*R*. This means that we can apply a refractive index change to compensate the thermal expansion. Denoting the observed HWHM of the Lorentzian profile in time as *t*_0_, it can be shown that the volume rate that must be applied to compensate the expansion is *V̇* = −3*V* ln ρ/(4*n*′*kRt*_0_). For *t*_0_ = 7.6 min, as found in Figure 4, and with the same parameters as above, we have *V̇* = 0.79 mL min^–1^. The volume of the chamber was continuously increased at this rate in the measurement of Figure 3 to compensate for the thermal expansion. Here, contrary to the previous measurement, a change in volume is simpler to implement than a change in wavelength. That is because here the applied rate of change is small and constant in time, which is simpler to implement using a syringe on a commercial syringe pump (WPI AL2000).

In future developments, compensation of the heating effect could also be accomplished via a steady wavelength increase or via a direct temperature stabilization of the sphere. On the latter option, we can be more quantitative regarding the stability required. For a temperature change, Δ*T*, we have μ = *nk*Δ*z̅*, with Δ*z̅* = α*z*Δ*®T*, where α is the thermal expansion coefficient. Equating this to the average phase shift obtained for a refractive index variation, we find the simple equivalence Δ*T* = Δ*n*/α. For aluminum (α = 20 × 10^–6^ K^–1^) and the value of our resolution (Δ*n* = 4.5 × 10^–9^), we find Δ*T* = 0.2 mK. Therefore, for the same performance, the temperature should vary significantly less than this value. Stability will be simpler to achieve using materials with a low thermal expansion coefficient. For example, with zerodur (α = 0.05 × 10^–6^ K^–1^), we find Δ*T* = 0.1 K. One might also take the route of treating the effect of heating via signal processing. For example, Fourier filtering could be applied to remove low frequency components.

On the other hand, we expect that the effect of the fraction of heat that goes to the air cannot be compensated. Indeed, in that case, the temperature increase has no reason to be uniform throughout the volume inside the sphere. If the effect if not uniform, the phase acquired by the field on a given path is no longer a simple function of its length and therefore cannot be compensated by a volume change. The relative amount of heat that goes into the material or into the air must depend on the properties of the material, in particular, its thermal diffusivity. For that reason, we think that most of the heat is conducted to the material in our case, as our sphere is made of aluminum, which has a high thermal diffusivity.

**Figure 5:**
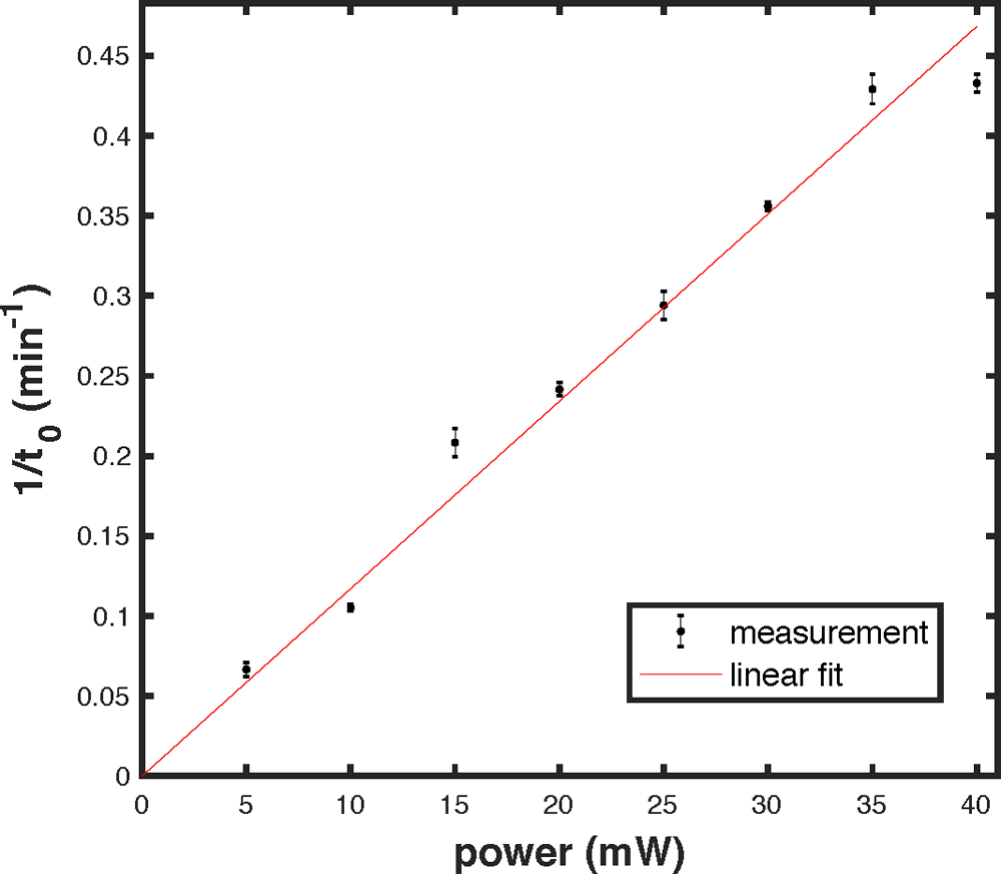
Effect of laser power on the rate of change of the speckle pattern. The inverse of the decay time is shown as a function of the input power. The center and span of the error bars, respectively, give the mean and standard deviation of 1/*t*_0_ in the duration of each measurement. We find an approximately linear relation with a proportionality constant of 0.012 min^–1^/mW, which supports the heating-related origin of the effect.

In order to further confirm the hypothesis that the slow change in the speckle is due to heating from the laser, we measure *t*_0_ for different powers of the input beam. Assuming again that the heat remains stored in the sphere’s material, we expect the inverse of *t*_0_ to be proportional to the input power. For each value of input power, we record the speckle patterns for 5 min and extract *t*_0_ by fitting with a Lorentzian profile. The result is shown in Figure 5, where we also display as error bars the standard deviation of 1/*t*_0_ during the 5 min of each measurement. We find an approximately linear relation reading 1/*t*_0_ = 0.012*P*, with *t*_0_ in minutes and *P* in milliwatts. From this we can infer the rate of change of the radius to be 0.1 nm·min^–1^·mW^–1^. We note in passing that, while presently undesired, in the future one could consider exploiting these effects to measure small changes in the temperature of the medium.

## Summary and Conclusion

In summary, we proposed a route to optimize speckle-based measurements of refractive index. While intuition suggests that the correct strategy is to maximize the path length of light in the medium of interest, it is more important to maximize the width of the path length distribution within the medium. In particular, we have demonstrated that an integrating sphere, in which light has a broad path length distribution, offers a simple yet sensitive probe of refractive index change of the medium it encloses. We quantified the change in the speckle pattern using the similarity (eq 1), analytically demonstrated that this takes a simple Lorentzian form as a function of refractive index change (eq 4), and verified it experimentally. We gave a general expression for the HWHM of the similarity curve, and found that it depends mainly on the radius and surface reflectivity of the sphere, which paves the way for possible optimizations. In our setup, we found the HWHM to be 6.5 × 10^–7^.

We exploited this high sensitivity to measure small refractive index variations of amplitude 4.5 × 10^–9^ with an uncertainty of 7 × 10^–10^. Our method allows a level of uncertainty comparable to the current state of the art techniques, but with a significantly simpler implementation. On the other hand, it allows the measurement of variations (instead of absolute values) in the refractive index and requires some care regarding heating effects due to the input laser light. We investigated this heating effect and found that 10 mW of laser power induces an increase in the sphere’s radius of 1.1 nm every minute by thermal expansion. This, however, can be compensated by applying either an appropriate volume or wavelength change and could also be reduced in future devices by a more judicious choice of the material from which the integrating sphere is constructed.

Importantly, the measurements presented here are 3 orders of magnitude more highly resolved than previous implementations based on laser speckle. Developments to the existing apparatus to improve both the temperature stability and the resolution will be guided by the progress made in state-of-the-art Fabry–Perot systems.^4^ In particular, the choice of material for the integrating sphere, plus the addition of active cooling, will significantly improve the thermal stability. Moreover, the use of shorter laser wavelength, larger image arrays and especially higher-reflectivity coating inside the sphere offer significant opportunities to measure even smaller refractive index changes. In the context of wavelength measurements, alternative forms of speckle analysis, such as principal component analysis^35^ and deep learning,^28^ have been shown to improve resolution and could also be applied to speckle refractometry. This work is most likely to find applications in chemical sensing, in particular, the detection of trace gases or small concentrations of chemicals in liquids. The study of the heating effect also suggests applications in the measurement of small temperature variations.
