# Correction to “Measurement of Variations in Gas Refractive Index with 10^–9^ Resolution Using Laser Speckle”

**DOI:** 10.1021/acsphotonics.2c01731

**Published:** 2022-11-16

**Authors:** Morgan Facchin, Graham D. Bruce, Kishan Dholakia

In the manuscript we incorrectly considered the inflection point of the Lorentzian similarity profile to be at the HWHM (half width at half-maximum) of the profile. More precisely, in the similarity profile given by
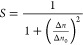
1(eq 4 in the main paper) where Δ*n*_0_ is the HWHM, the inflection point is not found at Δ*n* = Δ*n*_0_ as claimed, but at .

The purpose of using the inflection point was to maximize the slope. At Δ*n* = Δ*n*_0_, the slope is −1/(2*x*_0_) = −0.5/Δ*n*_0_. At Δ*n* = Δ*n*_0_, the true inflection point, the slope is . The slope at the true inflection point is about 1.3 times greater. For a given uncertainty on the measurement of *S*, the corresponding uncertainty on the refractive index variation decreases when the slope increases.

This does not change any conclusion of the manuscript. The point we used is simply not the optimal point. The uncertainty can be further reduced by a factor of 1.3.

